# Understanding the role of head size and neck length in micromotion generation at the taper junction in total hip arthroplasty

**DOI:** 10.1038/s41598-024-57017-x

**Published:** 2024-03-16

**Authors:** Federico A. Bologna, Giovanni Putame, Alberto L. Audenino, Mara Terzini

**Affiliations:** 1 PolitoBIOMed Lab, Politecnico di Torino, 10129 Turin, Italy; 2 Department of Mechanical and Aerospace Engineering, Politecnico di Torino, 10129 Turin, Italy

**Keywords:** Health care, Musculoskeletal system

## Abstract

Modular hip implants allow intra-operative adjustments for patient-specific customization and targeted replacement of damaged elements without full implant extraction. However, challenges arise from relative micromotions between components, potentially leading to implant failure due to cytotoxic metal debris. In this study magnitude and directions of micromotions at the taper junction were estimated, aiming to understand the effect of variations in head size and neck length. Starting from a reference configuration adhering to the 12/14 taper standard, six additional implant configurations were generated by varying the head size and/or neck length. A musculoskeletal multibody model of a prothesized lower limb was developed to estimate hip contact force and location during a normal walking task. Following the implant assembly, the multibody-derived loads were imposed as boundary conditions in a finite element analysis to compute the taper junction micromotions as the relative slip between the contacting surfaces. Results highlighted the L-size head as the most critical configuration, indicating a 2.81 μm relative slip at the mid-stance phase. The proposed approach enables the investigation of geometric variations in implants under accurate load conditions, providing valuable insights for designing less risky prostheses and informing clinical decision-making processes.

## Introduction

At their introduction, hip prostheses were *monobloc*, consisting of a singular part for the entire femoral component having fixed dimensional combinations with respect to the lengths of the stem, neck, and head diameter. Unfortunately, this design inhibited the precise customization of the implant to fit the patient’s anatomy, resulting in early failure^1,2^. Nowadays, surgeons can take advantage of the modularity of hip replacements, which allows intra-operative adjustment to adapt the prosthesis to the patient’s anatomy or to replace only the damaged element without the need to remove the entire implant^3^, making the replacement surgery faster and less risky for the patient. The concept of modularity suggests the potential of combining the components of the implant by selecting the most suitable parameters, such as neck length and head size according to the specific requirements of the patient^4^.

A crucial aspect of modular prostheses involves the coupling between the femoral head and the neck stem. Initially, this connection consisted of screwing the head to the neck. However, this approach was discontinued because the threaded connection tended to loosen or damage due to relative movements and loads borne by the hip joint. Hence, couplings were developed based on the Morse taper principle, which currently represents the most common adopted solution. It involves a tapered design where the trunnion of the neck stem (male portion) fits tightly into the tapered opening of the femoral head (female portion), creating a strong and stable connection after the application of a compression assembly load. The compression-based mechanism makes the Morse taper particularly compelling for operating under significant compression loads, such as those experienced, for instance, by hip or dental prostheses. Moreover, the absence of additional fasteners like screws or bolts eliminates potential points of failure, enhancing the longevity of the implant^1,2^, and simplifying the surgical technique, consequently, reducing risks related to the surgery.

Micromotions at the head-neck junction pose a critical issue in total hip arthroplasty (THA), as they account for up to 3% of implant failures and subsequent revisions^5^ due to the generation of cytotoxic metallic debris (*trunnionosis*)^6^. Although this percentage may seem low, it becomes significant when considering the total number of revision surgeries performed. As an example, there were 129127 revision hip arthroplasties performed in the U.S. between 2012 and 2022, 1577 revisions in Italy in 2020 alone, and 43682 in the U.K. from 2003 to 2022^7–9^. Different studies have investigated the key design parameters that impact micromotions^10,11^, revealing dependencies on the materials comprising the coupling^12^, assembly load^13,14^, head diameter^15,16^, extension of the contact surfaces^12,17^, and implant offset^18^. The adoption of material with a lower Young’s modulus for the head indeed increases the occurrence of micromotions between the head and stem, amplifying the risk of fretting corrosion^19^. Micromotions between the contacting surfaces are also affected by the entity of the assembly load. The majority of research suggests that an increase in assembly force results in improved prosthesis stability, reduced micromotions, and consequently, decreased fretting wear. The recommended force to achieve these benefits is 4 kN, which allows maintaining micromotions at an approximate average value of 3 μm up to 2 million cycles^14^. Excessive forces (15–17 kN) may potentially lead to femoral or prosthesis fractures and may not consistently yield optimal results, contributing to an increase in wear^20^.

A widely studied factor is the head diameter. On one hand, a larger head enhances the stability of the prosthesis, reduces contact pressures by expanding the load distribution area, and minimizes the risk of dislocation and impingement. On the other hand, it is linked to pronounced wear^16^. Extensively studied are the parameters influencing the dimension of the contact surface, as any deviation from the perfect fit configuration reduces the effective contact area, leading to the formation of clearance that facilitates micromotion between the contacting surfaces^12,17^. These parameters include the taper angle mismatch^21^, referring to the angular difference between the taper head and stem trunnion, and the variability introduced by manufacturing tolerances^4,22^.

Lastly, the implant offset completes the range of commonly considered parameters, which is the perpendicular distance between the diaphyseal axis of the femur and the center of rotation of the femoral head. The anatomical offset can be restored during hip replacement surgery by varying the head size, namely through a variation in the taper depth, and/or the neck length. The variation of the head size introduces the presence of a moment arm (or lever arm), which results in a greater load on the head-neck coupling^23^. The definition of the moment arm is not consistent in literature: Elkins *et al.*^15^ measure it as the distance between the center of the femoral head and the center of pressure on the trunnion, whereas Kao *et al.*^22^ define it as the distance between the center of the head and the point where the head taper and the trunnion stem come into contact. In this study, the moment arm is defined as the distance, along the longitudinal axis of the neck, between the head center and the contact center, which is the center of the trunnion portion contacting the head taper.

At a clinical level, achieving equal offsets can be accomplished by adjusting either the head size or the neck length. However, the intraoperative adjustment of the offset is limited to the selection of the head size which, in turn, leads to the generation of the moment arm. Numerous retrieval studies have suggested that the moment arm introduced by head size variation can have a significant impact on the onset of micromotions, which are strictly related to implant longevity^4^. In particular, Martin *et al.*^24^ assessed fretting and corrosion in a cohort of twenty-one explanted prostheses, indicating how corrosion may be exacerbated by large head sizes introducing moment arms equal to or greater than 4 mm. Nevertheless, poor literature has effectively isolated the effect of the moment arm in the generation of implant micromotions.

The few experimental studies on micromotions assessment at the taper junction uses eddy current sensors^19^ or surface topographies^18^ but, due to the complexity of measurement and associated costs, the assessment of micromotions between the stem and head is often conducted through finite element (FE) methods. FE analyses typically aim to predict wear related to micromotions^25,26^ at the taper junction using approaches of varying accuracy^27,28^, without explicitly isolating the impact of the moment arm resulting from variations in head size. However, these studies frequently encounter the limitation of solely predicting mechanical wear^29^, while electrochemical processes may have an additional effect on the wear of the taper interface, underestimating the actual volume of wear^30^.

In this context, the assembly force and subsequent in vivo loads are often applied at the center of the head and rigidly transmitted to its outer surface^13,15,18,31^. Typically, in FE studies, hip loads are usually applied at a fixed point^20,32^. This approach may introduce bias due to the constraint of the head center of rotation, whereas it should be able to readjust its location freely. In some studies, a remote reference point simulating the action of the stem is used^32^, while in others, the loads are applied at the base of the trunnion^33^. Furthermore, the center of the head is also utilized to apply the loads that the prosthesis undergoes during daily activities^20^. This discrepancy in the application of loading conditions could lead to a failure in accurately represent the reality of the implant work environment^27^. Additionally, micromotions are typically assessed through the resultant of the relative displacement between the contacting surfaces, losing the association with the slip direction, which is fundamental for an accurate wear estimation. As far as loads used for FE models are concerned, they are often derived from international standards (e.g*.*, ISO, ASTM) or experimental studies based on instrumented prostheses. In the first case, it has been shown that standards may underestimate or simplify in vivo loads^34,35^. In the second case, researchers are compelled to use the application points reported in the studies, such as the center of the femoral head. In this framework, musculoskeletal multibody (MB) models can serve as valuable tools to derive implant-specific loads and application points for transfer into the FE model^36–41^.

In this work, a combination of MB and FE approaches is proposed to estimate the taper junction micromotions for different geometric configurations of a parametrized hip prosthesis. The MB approach was initially employed to construct a musculoskeletal model of a lower limb and to derive the hip contact force and location during a gait cycle by varying the head size and neck length of the implant. Subsequently, the MB-derived loads were applied as boundary conditions in FE simulations to determine the taper junction micromotions, representing the relative slip between the contacting surfaces in terms of both magnitudes and directions, in order to provide more reliable information to be exploited in wear estimations. The novelty of this study lies in evaluating the impact on implant stability of each geometric parameter separately, without biases induced by boundary conditions or other hidden secondary factors. Specifically, the study sheds light on the role of the moment arm on the micromotions generation at the head-neck taper junction. Ultimately, this research aims to provide valuable insights for the design of prostheses with lower risk and for informing clinical decision-making processes.

## Methods

### Implant design and configurations

**Figure 1:**
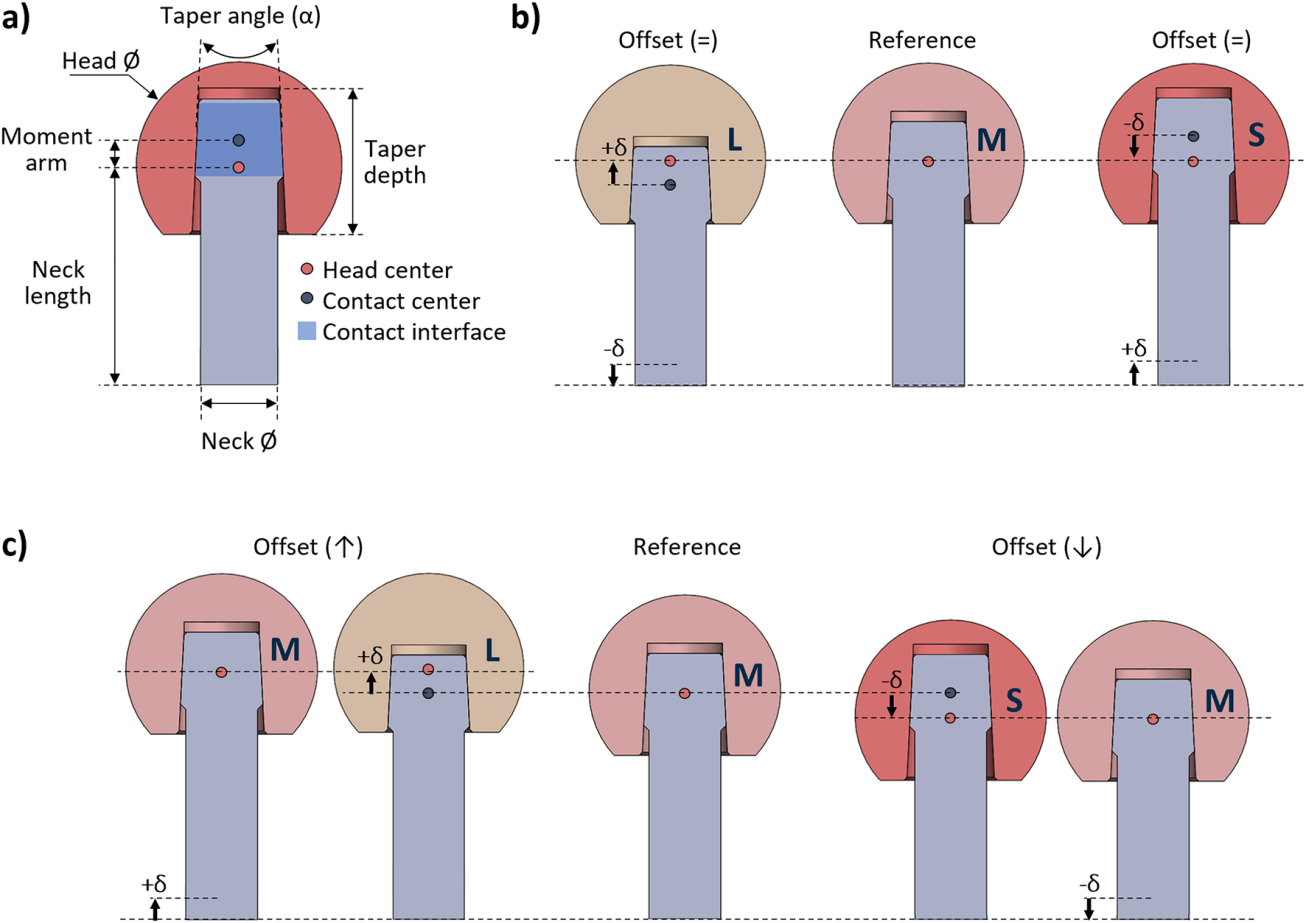
Head-neck junction geometry: (**a**) main considered geometric features of the coupled implant components; (**b**) junction configurations showing the same implant offset obtained by a concurrent opposite change of both head size and neck length; (**c**) junction configurations showing different implant offsets by a change of the head size through the moment arm or neck length (± δ = 4.0 mm). Capital letters refer to the head size (*S* small, *M* medium, *L* large), black arrow indicates the parameter variation. Note: the moment arm is null when the head center and contact center are coincident.

The head-neck junction geometry of the implant was designed in SolidWorks 2022 (Dassault Systèmes, Vélizy-Villacoublay, France). It comprises two components representing a neck with its trunnion and a femoral head (Fig. 1a). The so-called 12/14 taper standard was selected for its wide usage in hip implants. Although ‘12’ and ‘14’ represent the diameters in millimeters at the top and bottom of the trunnion, respectively, the taper geometry is not uniform and varies depending on the implant manufacturer^4,29^. In detail, according to the standard, an angle (α) of 5.7° was chosen for both the trunnion and head taper without mismatch, namely, assuming a perfect fit coupling. This assumption was adopted to maintain a consistent contact across the entire head-neck interface, thereby excluding the contribution of a variable contact area on the simulation results. Furthermore, based on common sizes provided by implant manufacturers and reported in similar studies, a diameter of 32.0 mm, with a clearance of 0.1 mm relative to the acetabular cup, was selected for the femoral head, while the neck stem was chosen to have a constant diameter of 12.0 mm^35,42,43^. Starting from these dimensions, a reference configuration of the junction was defined by setting a M-size head (with a taper depth of 18.7 mm) and a neck length of 38.0 mm. No moment arm was implemented for this reference configuration, meaning that the head center and the contact center are coincident. Detailed drawings of the head-neck junction are reported in the supplementary material (Supplementary Fig. S1).

Then, the reference configuration was modified by varying the taper depth (*i.e.*, head size) and/or the neck length of *δ* = ±4.0 mm, leading to six additional configurations of the head-neck junction. The considered variations of the parameters allowed to stay within the ranges of sizes indicated by manufacturers, while maintaining the trunnion completely inside the head taper despite the investigated implant configurations, thus avoiding variation in the head-neck contact area. Specifically, two additional L-size and S-size heads were considered, having a taper depth of 14.7 and 22.7 mm, respectively. On the other hand, values of 34.0 and 42.0 mm were considered for the neck length variation. With respect to the reference, the additional configurations can present an unvaried (30.5 mm), increased (33.7 mm), or decreased implant offset (27.3 mm). Indeed, the change of only one parameter causes an offset variation (Fig. 1c). For instance, an increase in the neck length as well as a greater head size leads to a corresponding increase in the implant offset. Conversely, a concurrent opposite change of both parameters results in the same offset (Fig. 1b). For instance, an increase in neck length along with a smaller head size does not produce a variation of the implant offset. However, it is noteworthy that not all the alternative configurations resulting in the same implant offset involve the presence of a moment arm. For example, a smaller head size or, alternatively, a decrease in the neck length can produce the same decrease in the implant offset, although only the first option entails the generation of a moment arm.

### MB model of the lower limb

**Figure 2:**
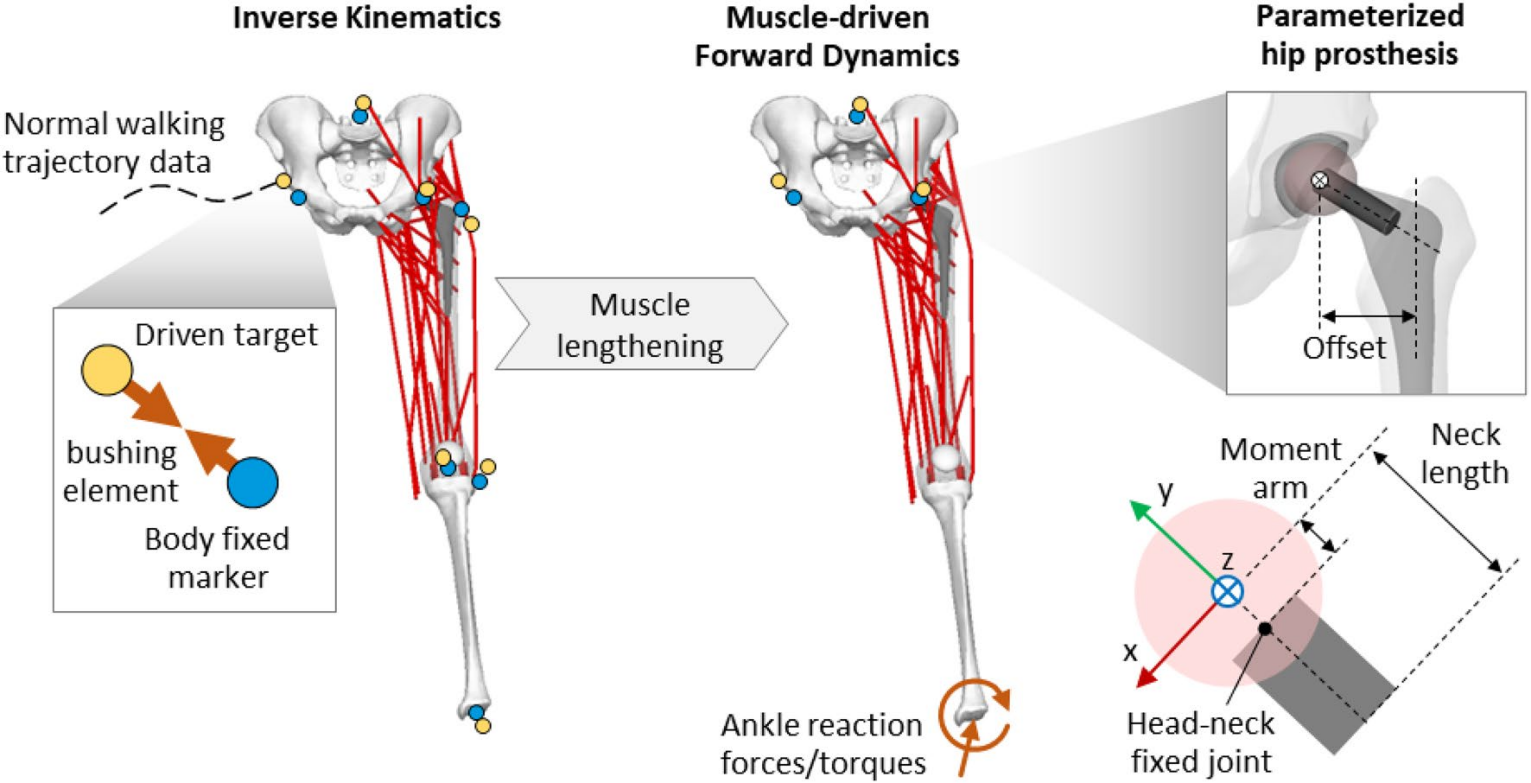
Parametric multibody model used for estimating the hip contact forces and contact location on the femoral head during normal walking.

A MB model of the left lower limb (Fig. 2) was developed using Adams software 2017 (MSC Software Corporation, Santa Ana, USA) to estimate the hip contact force (HCF) and contact point location during a normal walking task. The model comprises bony geometries from the pelvis to the tibia, major hip muscles, and a parametrized hip prosthesis.

In detail, standardized bone geometries (Sawbones Europe AB, Malmoe, Sweden) were scaled according to data of an average patient from the OrthoLoad dataset with a body weight (BW) of 836 N^44^. In addition, the patella was represented by an ellipsoid with congruent dimensions. The femur and tibia masses were computed as percentage of the total BW, resulting in 8.9 kg (10.5%) and 3.8 kg (4.5%) for femur and tibia, respectively^45^. The parametrized hip prosthesis comprises a cylinder representing the implant neck, extending from the osteotomy level to the center of the femoral head. The femoral head is modeled as a sphere centered within a simplified acetabular cup, which, in turn, is represented as a hollow hemisphere rigidly fixed to the pelvis. Thus, a contact pair was defined at the hip joint, modeled using an impact formulation^37^ with a contact stiffness of 2.7 × 10^6^ N/mm^1.5^ calculated based on the Hertzian theory, considering the material properties (Table 1) of a femoral head of zirconia-toughened alumina (ZTA) and an acetabular cup of ultra-high molecular weight polyethylene (UHMWPE). Based on this formulation, the contact point is defined as the centroid of the intersecting volume between the undeformed shapes of the head and the acetabular cup, which compenetrate during the contact phase.

**Table 1:** Material properties for implant components.

Component	Material	Young’s modulus	Poisson’s ratio
Femoral head	ZTA^6^	E_1_ = 350 GPa	ν_1_ = 0.23
Neck stem	Ti6Al4V^6^	E_2_ = 110 GPa	ν_2_ = 0.32
Acetabular cup	UHMWPE^46^	E_3_ = 700 MPa	ν_3_ = 0.46

Concerning the knee joint, a one-degree-of-freedom (DoF) hinge joint was employed, with the patella constrained to move, together with the tibia, along a circular pathway around the intercondylar axis, which was defined as the axis passing through the centers of two ellipsoids inscribing the femoral condyles.

The major muscles crossing the hip joint (Supplementary Table S1) were included in the model with origin and insertion attachment points adapted from the ‘Gait2392’ model provided by the OpenSim software^47^. Each muscle bundle was implemented as an actuator applying traction force between the origin and insertion points, and via-points were added to account for non-linear muscle paths (e.g*.*, gluteal muscles). Furthermore, a torque actuator located at the knee joint was used to compensate for the absence of the gastrocnemius muscle and its contribution to knee flexion.

Overall, the MB analysis involved two sequential steps. Briefly, first, an inverse kinematics of 1.1 s was carried out where the motion is prescribed to the body segments of the model by using a set of *motion agents* each consisting of a body-fixed marker coupled, through a bushing element, to a target driven by markers’ trajectories obtained via motion capture of a normal walking task^44^ (Fig. 2). Secondly, the resulting muscle lengthening was recorded and used as input to a muscle-driven forward dynamic simulation, where external forces and torques are directly applied to the ankle joint while the pelvis kinematics was prescribed. The muscle action, required to reproduce the previously recorded muscle lengthening patterns, was computed by feedback controllers and limiting the generated force to the maximum isometric force (Supplementary Table S1) of each muscle^47–50^.Finally, the two-step MB analysis was repeated by varying the prosthesis neck length, head size, or both of them as previously described. In particular, in the MB framework, the head size variation turns out in a moment arm change obtained by moving a defined fixed head-neck joint along the neck axis. Thus, the resultant HCF and contact location with respect to a local reference system centered on the femoral head were computed. After filtering in Matlab R2021b (MathWorks, Natick, Massachusetts, USA) by using a fourth-order low-pass Butterworth filter with a cut-off frequency of 10 and 30 Hz for contact force and contact location, respectively, the derived data were compared and used as boundary conditions for subsequent FE models of the taper junction.

The performances of the MB model were validated by comparing the trend and peak of the resultant HCF, computed for the reference configuration, with data reported in similar numerical studies and measurements provided in the OrthoLoad dataset^44^.

### FE model of the taper junction

**Figure 3:**
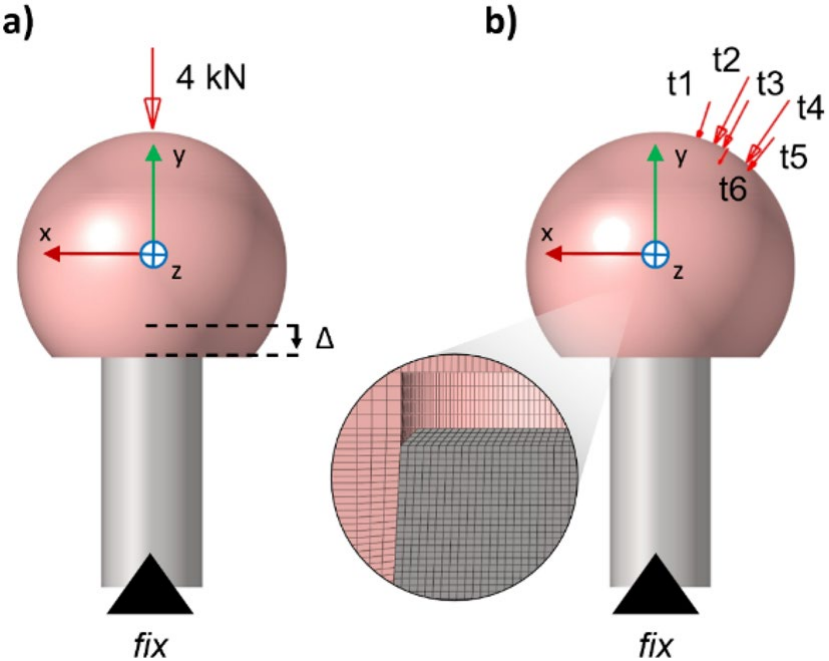
The two phases of the FE model are represented for the reference configuration. (**a**) Application of the assembly load and longitudinal displacement of the head (*Δ*); (**b**) Application of the MB-derived load in six time steps. The distal end of the trunnion was fixed (black triangle) in both phases.

The head-neck junction geometry was imported into Abaqus/CAE (Dassault Systèmes, Vélizy-Villacoublay, France) for the implementation of the FE model. The material properties listed in Table 1 were assigned to the head (ZTA) and to the trunnion (Ti6Al4V). Both materials were considered linear and isotropic. The geometry of the two components was divided into *sweepable* portions in order to discretize them using 8-node linear brick elements (C3D8R). As a result of the mesh convergence study, an element size of 0.2 mm was adopted in the contact region of the taper junction. In detail, the mesh element size was progressively decreased, assuming that results were mesh-independent when the difference between the solution of two consecutive mesh refinements was less than 2% in terms of both longitudinal displacement of the head (*Δ*) and average contact pressure of the trunnion (*p̄*), as reported in the supplementary material (Supplementary Fig. S2). The contact interaction at the taper junction was modeled as *finite sliding* setting the *penalty* contact formulation and a constant isotropic friction coefficient (*μ*) of 0.21^31^. The trunnion base was fixed in all DoF to simulate the locking effect with the femoral bone expected to occur after inserting the stem into the femoral diaphysis. The FE analyses were divided into two phases: (1) application of the assembly load and (2) application of the MB-derived loads involved in the gait cycle. In the initial phase, an assembly load of 4 kN^31^ was applied in an implicit static simulation to the center of the upper surface of the head, parallel to the taper axis (Fig. 3a). The final steps of the assembly phase were introduced with zero load to account for a recovery phase.

An analytical verification of the parameters employed for the mesh convergence analysis was performed. The average contact pressure (*p̄*) was estimated as a function of the assembly load using the following equation^51^:

1 $$\overline{p }=\frac{{F}_{a}}{A(\mu {\text{cos}}\left(\alpha /2\right)+{\text{sin}}(\alpha /2))}$$

where *F*_*a*_ is the static axial assembly force, *A* is the contact area (476.9 mm^2^), and α is the taper angle. Finally, the longitudinal displacement of the head (*Δ*), considering axial deformations, was verified using the following equation^51^:

2 $$\Delta =\overline{p} \, r \, \mathrm{cot }(\alpha) \left\{\frac{\left(1-{\nu }_{1}\right)}{{E}_{1}}+\left(\frac{{R}^{2}+{r}^{2}}{{R}^{2}-{r}^{2}}+{\nu }_{2}\right)/{E}_{2}\right\}$$

where *R* and *r* are the radii of the head and trunnion, respectively, at the contact center.

The second phase of the FE analysis was conducted to simulate the effect of the normal walking cycle (Fig. 3b). An implicit static simulation was implemented sequentially applying the MB-derived loads at their specific contact point on the head surface as consecutive ramp loads. For this purpose, six consecutive time instants (t1–t6) were considered, with two instants (t2 and t4) corresponding to the highest peak values of the resultant HCF, while the others were arbitrarily chosen at about 6, 33, 59, 73% of the walking cycle to reproduce the overall force trend.

To investigate the impact of the load application method, a comparative model was created by applying the loads at the center of the head and rigidly transmitting them to its outer surface^13,15,18,31^ (Supplementary Fig. S3). In this case, a single implicit static simulation of the same duration as the MB simulation was conducted taking into account the MB-derived reaction torques measured at the head-neck fixed joint.

The FE analyses were performed by using the Abaqus/Standard implicit solver on twelve computing cores of a workstation equipped with Intel^®^ Core^™^ i7-12700 and 32 GB RAM. Micromotions between taper and trunnion were evaluated in HyperView (Altair Engineering, Troy, MI, USA) as the relative slip between the contact surfaces (CSLIP). At the end of each step (subscript “tn”), the *CSLIP* value obtained at the end of the assembly step (subscript “ta”) was subtracted, as reported in Eq. (3).

3 $$|{CSLIP}_{n}|=\sqrt{{{(CSLIP1}_{tn}-{CSLIP1}_{ta})}^{2}+{{(CSLIP2}_{tn}-{CSLIP2}_{ta})}^{2}}$$

Finally, the instantaneous tangent directions of the contact slip (*CTANDIR*) were recombined to calculate the vectorial components of slipping (*CSLIP*) in cylindrical coordinates as follows:

4a $${CSLIP}_{x}={(CSLIP1}_{tn}-{CSLIP1}_{ta})CTANDIR1.x + {(CSLIP2}_{tn}-{CSLIP2}_{ta})TANDIR2.x$$

4b $${CSLIP}_{y}={(CSLIP1}_{tn}-{CSLIP1}_{ta})CTANDIR1.y + {(CSLIP2}_{tn}-{CSLIP2}_{ta})TANDIR2.y$$

4c $${CSLIP}_{z}={(CSLIP1}_{tn}-{CSLIP1}_{ta})CTANDIR1.z + {(CSLIP2}_{tn}-{CSLIP2}_{ta})TANDIR2.z$$

This operation was implemented in HyperView using the *VectorFromScalar* function.

## Results

### Hip contact force and location

The obtained resultant HCF for the reference configuration is comparable with those reported in similar numerical studies^41,52^, although an overestimation of 351 N (0.42 BW) can be observed for the peak force with respect to the experimentally measured reference data^35,44^, as shown in the supplementary material (Supplementary Fig. S6).

**Figure 4:**
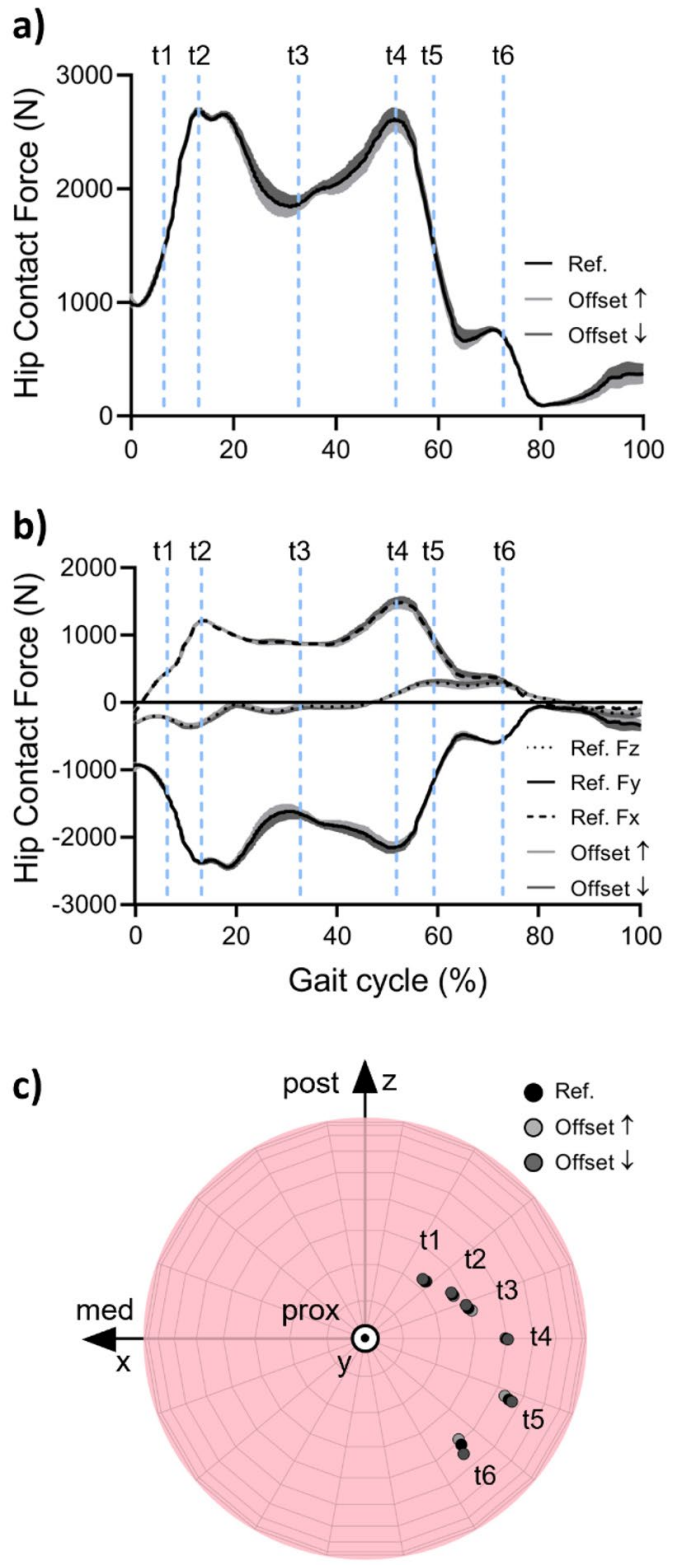
Hip contact force and location resulting from MB simulations by varying the implant offset. (**a**) Force resultant, (**b**) force components, and (**c**) contact points.

From the estimated resultant HCF, a typical curve characterized by two peaks can be observed in all performed simulations. Specifically, for the starting reference configuration a maximum resultant HCF of 2679 N (3.2 BW) was computed at the first peak in the gait cycle (Fig. 4a), which remained almost unvaried (max variation < 29 N) after changes in prosthesis parameters. Conversely, the greatest resultant HCF alteration was observed at the characteristic second peak of the gait cycle, corresponding to the terminal stance phase. The observed force variation rises from 2604 N up to 2697 N (+3.5 %) in case of shorter implant offsets, while it decreases to 2516 N (−3.4 %) for longer offsets. Looking at the force components (Fig. 4b), it can be derived that the HCF alteration belongs mainly to the y-component of the force (directed along the femoral neck axis), which shows the highest force values followed by the x-component. The z-component, perpendicular to the coronal plane, is not importantly affected by the prosthesis variations.

With reference to the contact location on the femoral head during the stance phase of the gait cycle (Fig. 4c), a negligible alteration in the contact point trajectory was observed among the different configurations (Supplementary Table S2). In general, the contact point moves anteriorly along an arc path located apically on the femoral head roughly surrounding the y-axis of the local reference system.

Moreover, it is worth mentioning that the variation in the head size, as implemented in the MB framework, does not affect the computed HCF since the overall prosthesis geometry is not modified as well as the relative orientation of the used local reference system. Contrarily, differences are generated in the reaction torques computed at the head-neck fixed joint. Indeed, moving the joint along the neck axis implies a moment arm alteration and, in turn, a reaction torque variation, especially around the z-axis of the head (Supplementary Fig. S4).

### Taper junction micromotions

The FE model of the taper junction exhibited an average contact pressure (*p̄*) on the trunnion surface of 23.97 MPa at the end of the assembly phase, while the average longitudinal displacement of the head (*Δ*) was equal to 34.05 μm. The error of the analytical calculations compared to the FE model results was below 1.5% for both quantities.

**Figure 5:**
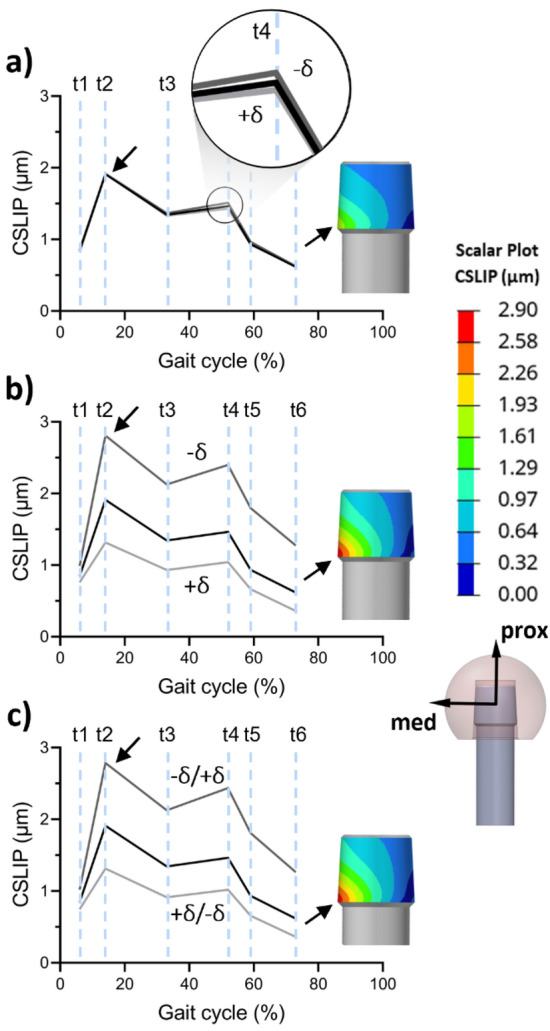
Maximum contact relative slip (CSLIP) at each time step with the black line representing the reference configuration: (**a**) neck length variation; (**b**) head size variation; (**c**) combined parameters variation.

In Fig. 5, the results obtained at the end of the application of MB-derived loads on the head surface are presented. The reference configuration exhibited a maximum CSLIP of 1.91 μm in the distal-medial area of the trunnion at 14% (t2) of the gait cycle (*i.e.*, the start of the mid-stance phase). Negligible changes were observed due to neck length variation (Fig. 5a), for which the maximum difference with the reference curve was found at 52% of the gait cycle (t4) for the short neck and corresponded to around 3% of the reference value at the same time step. In contrast, the variation in head size (Fig. 5b) led to considerable changes in micromotions. While a smaller head size (S-size) resulted in a reduction of the maximum CSLIP to 1.32 μm, a greater head size (L-size) led to a maximum CSLIP of 2.81 μm. In addition, the combined variation of both parameters to maintain the same implant offset (Fig. 5c) resulted in comparable outcomes to those with the only variation in head size. In this scenario, the maximum CSLIP was consistently in the same location with a value of 2.79 μm. Furthermore, micromotions were determined for the reference configuration by applying the MB-derived forces and torques at the center of the head. Under such boundary conditions, the CSLIP peak previously computed at t2 decreases by approximately 20%, underestimating the micromotions at the conical junction. A detailed comparison of outcomes deriving from the two alternative boundary conditions is provided in the supplementary material (Supplementary Fig. S5).

**Figure 6:**
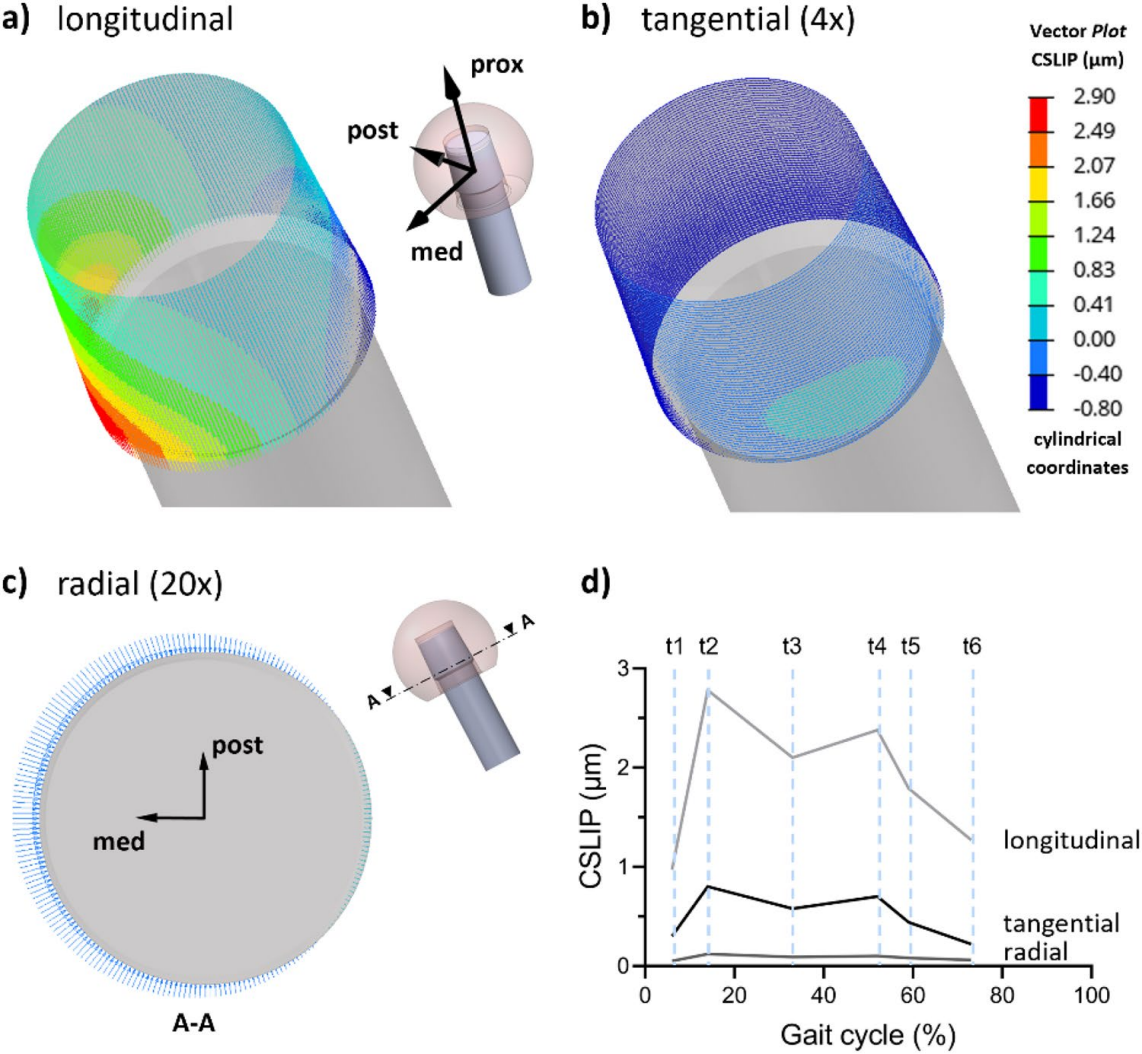
CSLIP for the L-size head configuration at t2 in cylindrical coordinates: (**a**) longitudinal component; (**b**) tangential component (4 × magnification); (**c**) radial component (20 × magnification) of the trunnion transversal section (A-A); (**d**) graph of the maximum values of the three components.

Finally, Fig. 6 depicts the vector plot of the CSLIP for the configuration with the L-size head at t2. From the calculation of the single components of the CSLIP in cylindrical coordinates, it is possible to observe that the longitudinal component (Fig. 6a) is predominant in this time step (2.78 μm) and it is located in the distal-medial area of the trunnion. The maximum value in the tangential component (Fig. 6b) was equal to 0.80 μm, localized in the medial-posterior distal area. In the same time step, the maximum radial compression of the trunnion is also localized (Fig. 6c) near the peak of the longitudinal component, corresponding to 0.12 μm. The maximum values of the three components are reported in the graph in Fig. 6d.

## Discussion

The necessity of integrating specific boundary conditions in the numerical investigation of hip prosthesis behavior is confirmed by several studies in the literature. Indeed, Feyzi *et al.*^28^, in their review on the FE simulation of fretting wear and corrosion in the taper junction, suggest the use of loading derived from daily living activities instead of simplified conditions as those reported, for instance, in ISO standards. To address this necessity, a MB model of the prosthesized limb was developed able to provide HCF and contact location on the femoral head.

The resulting HCF obtained for the reference configuration aligns with findings from similar numerical studies^41,52^, with an overestimation of peak forces compared to experimentally measured reference data^35,44^. Furthermore, the MB model allowed for varying the implant parameters, namely, head size and neck length, resulting in the eventual increase or decrease of the implant offset. In this regard, simulations with short offsets led to higher HCF than the reference configuration, and *vice versa*. This behavior is expected, as shorter offsets result in a reduction of the moment arm for muscles spanning the hip joint. Consequently, higher muscle forces, and hence higher intra-articular contact forces, are required to generate the same torque necessary for achieving a specific joint kinematics^53^. In the literature, a HCF reduction of 5.7% was reported for an offset variation of +4.8 mm^54^, which aligns with the findings of this study (3.4%), considering that in the presented MB model a neck length reduction of −4.0 mm involves a variation of −3.2 mm in offset. In other studies, similar results are reported as effect of acetabular cup medialization that corresponds, in turn, to an implant offset increase. Specifically, a 5.0 mm cup medialization produced a reduction in HCFs between 4.4% and 6.6% compared to the anatomical offset^55,56^. Conversely to the HCF, which were directly measured in vivo thanks to the development of instrumented implants ^35^, to date, the trajectory of the hip contact point during daily activities can be only computed by using musculoskeletal models. In this regard, looking at the contact location computed in this study for the stance phase, an arc path located apically in the anterolateral region of the head was observed, which is qualitatively in agreement with the literature^34,57,58^. Also, it was observed that different offsets led to similar contact trajectories, according to findings reported by De Pieri *et al.*^56^. As far as concerned the clinical functional outcomes, the same authors associated the computed contact trajectory with the region where the most polyethylene wear of the acetabular cup occurs in vivo.

Using the FE approach, the MB outcomes were exploited to investigate how alterations in the head size and neck length of the hip prosthesis can affect the stability of the taper junction. Primarily, the analytical verifications confirmed the reliability of the implemented FE model at the end of the assembly phase. Upon completing the assembly phase, English *et al.*^31^ achieved a maximum CSLIP of 9 μm employing a perfect fit configuration in a coupling between a CrCo head and a Ti6Al4V stem. Haschke *et al.*^19^ validated numerical outcomes between the head and stem using six eddy current sensors, obtaining micromotions ranging from 2.2 to 2.8 μm, which is close to the maximum value of the present study (1.91 μm in the reference configuration). However, this result was achieved with an assembly load of 2 kN and a CoCr29Mo head. The same head material was considered by Falkenberg *et al.*^18^ in a combined numerical and experimental work on tip fit configurations, revealing that smooth and micro-grooved stem tapers exhibited equal amounts of micromotion. In their work, it was found that large head sizes and low assembly forces significantly increased micromotions from 2.7 μm to 9.3 μm and from 4.1 μm to 8.8 μm, respectively. Similar experimental results were obtained by Mali and Gilbert^59^, in which fretting corrosion at the head-neck interface was associated with micromotions ranging from 5 to 12 μm. In addition, the use of material with a lower Young’s modulus for the head increases the occurrence of micromotions between the head and stem, thereby amplifying the risk of fretting corrosion^19^. The distal-medial area (*i.e.*, closest to the lesser trochanter) is confirmed to be the most critical region, as supported by the retrieval study of Martin *et al.*^24^. In accordance with the results reported by Elkins *et al.*^15^, at peak values of joint contact load, the CSLIP was predominantly parallel to the longitudinal axis of the trunnion.

From the obtained results, it was evident that the variation in head size, and therefore the introduction of a moment arm, significantly influenced the micromotions, conversely to the neck length variation, where, at the same moment arm, no important differences were observed compared to the reference configuration. From a different perspective, findings revealed how the implant offset variation due to different neck lengths, with consequent alterations of the hip contact force and location, appears less critical than the moment arm introduction. This outcome was consistent with the study by Arnholt *et al.*^12^, in which it was reported that the increase in the moment arm resulted in an increase in the bending moment and consequently in fretting corrosion. Furthermore, it is important to highlight the surgical implications of this aspect, as adjustments are often constrained to altering only the head size^60^. Nonetheless, even a slight increase in the HCF arising from a decrease in implant offset should be considered due to its implication in potentially accelerating implant wear over the long term, as in case of young patients undergoing early surgery.

The additional investigation conducted using a fixed point at the center of the head for the application of external loads varying the head size resulted in a boundary conditions bias, hindering the effect of the moment arm. Thanks to the MB model external loads were therefore applied at their specific contact point on the head surface, enabling the free readjustment of the center of rotation.

As a matter of fact, this study presents some limitations. The implemented MB model, although efficient in terms of computational costs, overlooks muscle activation dynamics with possible related co-contraction phenomena. Nevertheless, the chosen strategy for performing forward dynamics was deemed appropriate for the specific objectives of this study, which focuses on estimating customized load conditions and overcoming the investigation of muscle dynamics.

Regarding the FE analysis, the only perfect fit configuration was implemented which could be considered as the most stable condition^23,32^. It should be noted that in tip/base fit conditions, micromotions would be amplified by the reduction of the contact surface involving the variation of the contact center, hence, introducing a moment arm. Also, wear effects on the interface geometries were not taken into account since a condition immediately following implantation was investigated. Actually, the progression of implant wear will increase the micromotions present at the interface over time. Eventually, larger variations of the two design parameters under consideration (e.g*.*, +10 mm) could be adopted, as also documented in other study^24,61^. However, the proposed variations were deemed sufficient to appreciate the influence of these parameters. Another simplification arises from the static application of the assembly load^18^. This assumption was adopted to reduce the computational cost of the simulations. Future work will be conducted to integrate the assessment of linear and volumetric wear rates at the taper junction by employing Archard’s wear law^62^.

In conclusion, the present study reaffirms the findings of retrieval studies, highlighting the criticality associated with the L-size due to the generation of a moment arm between the center of the femoral head and the center of pressure on the trunnion. MB and FE simulations were combined to provide a more realistic representation of the complex biomechanical behavior of a hip prosthesis during a walking task, yielding more comprehensive insights into the loading conditions experienced by the implant. Overall, it should be noted that with this study a detailed drawing of a taper junction is provided, which can constitute a common benchmark for future similar works or round-robin studies. Following a thorough analysis of boundary conditions, the relative sliding between the contacting surfaces was determined in both magnitude and directions, providing more accurate data for wear estimation. Finally, this study emphasizes concerns related to the importance of selecting the combination of implant components that, although resulting in equal offsets, can reduce the amount of micromotions, thus minimizing the risk of critical wear.

### Supplementary Information

Supplementary Information.

## Data Availability

The majority of the data generated and analyzed during this study is included in the published article and its supplementary file. Any additional data are available from the corresponding author upon reasonable request.
