# Supplementary material for: Understanding the role of head size and neck length in micromotion generation at the taper junction in total hip arthroplasty
Source: Sci Rep. 2024 Mar 16;14:6397. doi: 10.1038/s41598-024-57017-x (PMC10944531; doi:10.1038/s41598-024-57017-x)
Supplement: Supplementary file 1 — Supplementary Information. [file 41598_2024_57017_MOESM1_ESM.pdf]

Understanding the role of head size and neck length in micromotion generation at the taper junction in total hip arthroplasty

Federico A. Bologna[1,2+], **Giovanni Putame**[1,2+], **Alberto L. Audenino**[1,2], **and Mara Terzini**[1,2*]

[1]Polito[BIO]Med Lab, Politecnico di Torino, Turin, 10129, Italy
[2]Department of Mechanical and Aerospace Engineering, Politecnico di Torino, Turin, 10129, Italy

[*]mara.terzini@polito.it
[+]these authors contributed equally to this work

Supplementary Material

Taper junction drawing

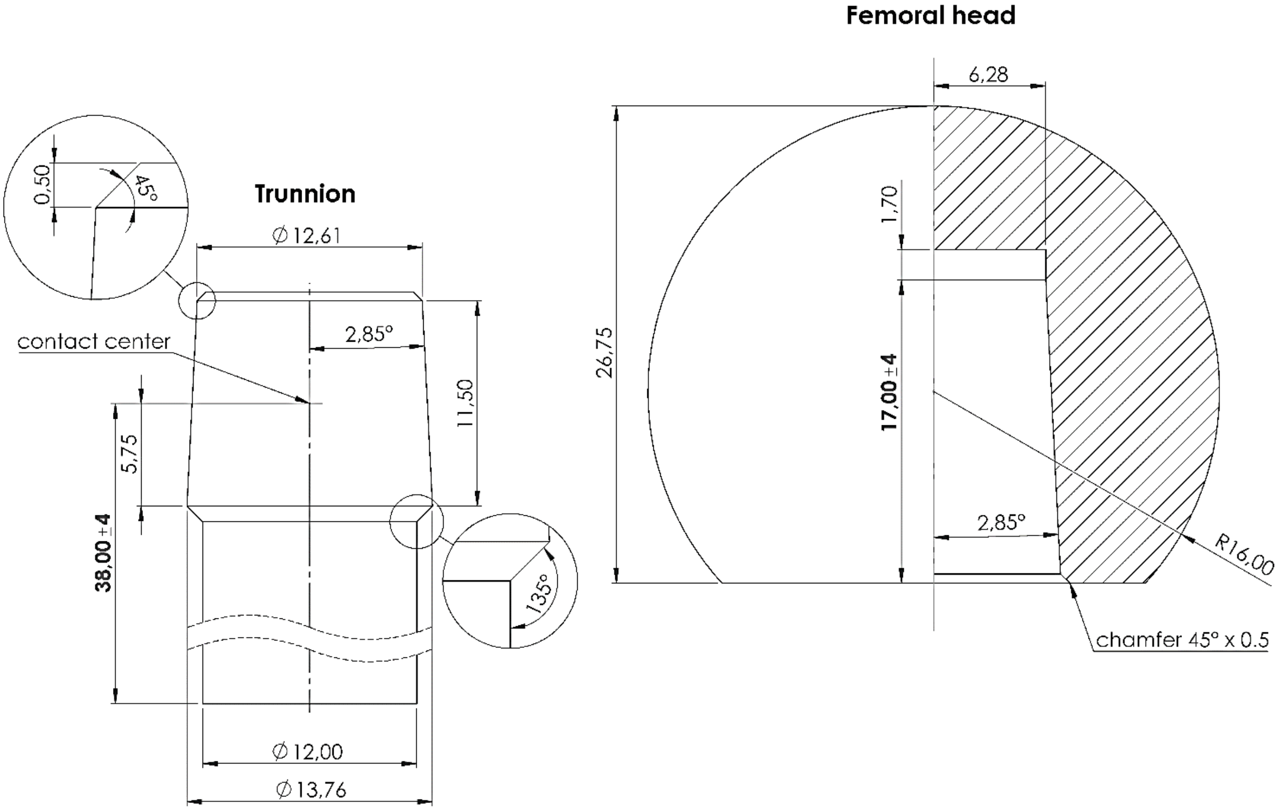

Figure S1. Detailed technical drawing of the trunnion component, on the left, and femoral head (semi-section view) of the femoral head.

Multibody model muscles

Table S1. Muscle bundle included in the multibody model with the relative maximum isometric force derived by the 'Gait2392' model provided by the OpenSim software[1].

Muscle	Bundle	Max isometric force (N)
Adductor brevis	1	429
Adductor longus	1	627
Adductor magnus	1	381
Adductor magnus	2	343
Adductor magnus	3	488
Biceps femoris brevis	1	804
Biceps femoris longus	1	896
Gemelli	1	164
Gluteus maximus	1	573
Gluteus maximus	2	819
Gluteus maximus	3	552
Gluteus medius	1	819
Gluteus medius	2	573
Gluteus medius	3	653
Gluteus minimus	1	270
Gluteus minimus	2	285
Gluteus minimus	3	323
Gracilis	1	162
Iliacus	1	1073
Pectineus	1	266
Piriformis	1	444
Psoas	1	1113
Quadratus femoris	1	381
Rectus femoris	1	1169
Sartorius	1	156
Semimembranosus	1	1288
Semitendinosus	1	410
Tensor fasciae latae	1	233
Vastus intermedius	1	1365
Vastus lateralis	1	1871
Vastus medialis	1	1294

Mesh convergence analysis

In the mesh convergence analysis, the element size in the contact region was progressively reduced. Starting from a dimension of 0.8 mm, the mesh size was gradually reduced until reaching 0.15 mm. The criterion for achieving mesh independence was considered met when the difference between the solutions of two consecutive mesh refinements was less than 2% in terms of both longitudinal displacement of the head (Δ) and average contact pressure of the trunnion ($\bar{p}$), as illustrated in Figure S2. As a result of the mesh convergence study, an element size of 0.2 mm was adopted in the contact region of the taper junction.
The two parameters analyzed in the mesh convergence were analytically verified[2] at the end of the assembly load, obtaining the following values:

- average contact pressure on the trunnion surface ($\bar{p}$) equal to 24.01 MPa;
- longitudinal displacement of the head (Δ) equal to 34.54 µm.

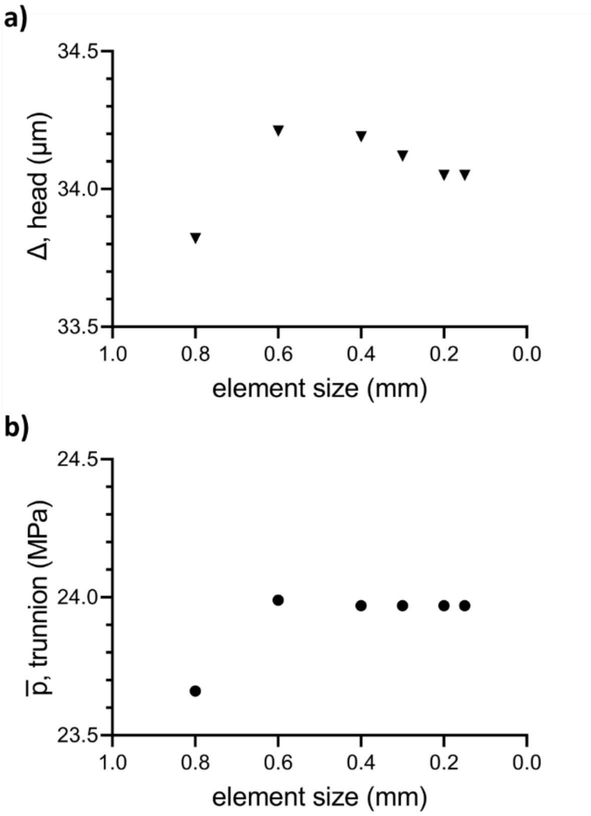

Figure S2. Outcomes of the mesh convergence analysis of the analytically verified parameters: a) longitudinal displacement of the head (Δ) and b) average contact pressure of the trunnion ($\bar{p}$).

Comparative FE model

To investigate the impact of the load application method, a comparative model was also created by applying the loads at the center of the head and transmitted rigidly to its outer surface[3–6] (Figure S3). In this case, a single implicit static simulation of the same duration as the MB simulation was conducted taking into account the MB-derived reaction torques measured at the head-neck fixed joint (Figure S4). Under this boundary condition, the CSLIP peak calculated for the reference configuration at t2 decreases by approximately 20% compared to the model where loads are applied to the head surface, thereby underestimating micromotions at the taper junction (Figure S5).

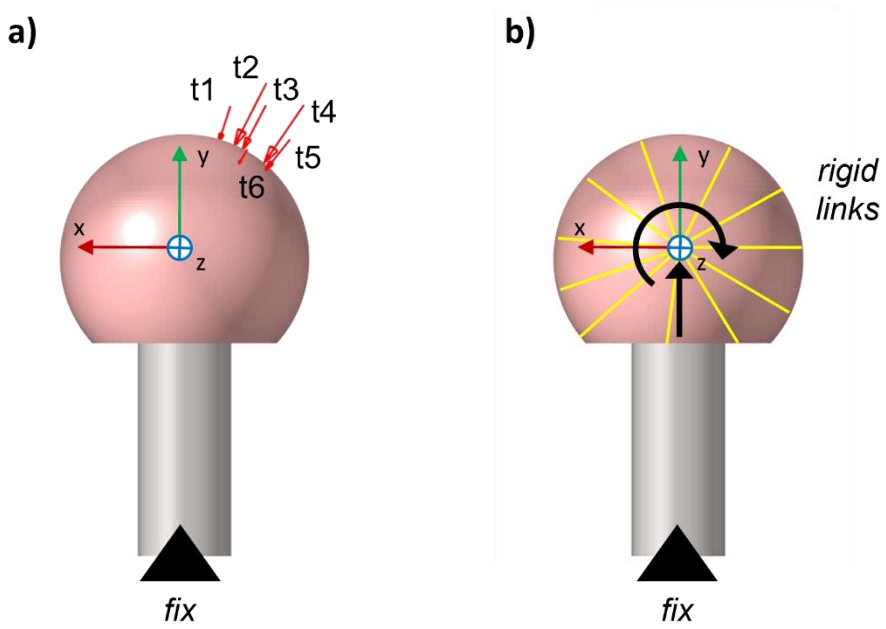

Figure S3. Comparative FE models are depicted for the reference configuration: a) Application of the MB-derived load in six time steps on the head surface. b) Application of the MB-derived reaction forces and torques at the head center. The distal end of the trunnion was fixed (black triangle) in both models, while yellow lines represent rigid links.

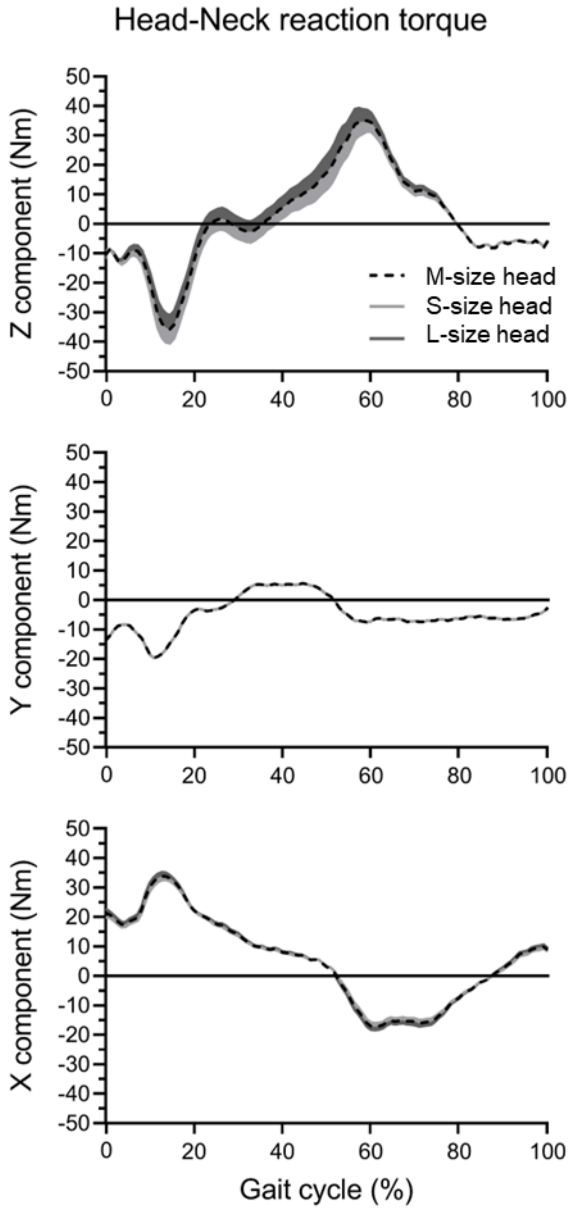

Figure S4. Components of the multibody-derived reaction torques measured at the head-neck fixed joint by varying the head size and maintaining the same implant offset.

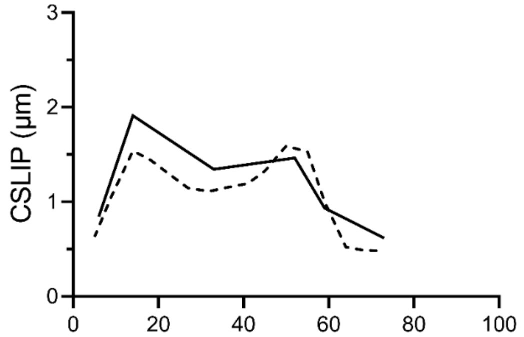

Figure S5. Comparison of the Maximum Relative Contact Slip (CSLIP) for the reference configuration obtained for the two FE models. The solid line represents the results obtained by applying loads derived from MB on the head surface, while the dashed line represents the results obtained by applying MB-derived loads at the center of the head.

Hip contact location

Table S2. Cartesian coordinates (mm) of the contact points on the femoral head at the considered instants (t1-t6) by varying the implant offset.

	**Offset ↓**			**Ref.**			**Offset ↑**		
Instant	x	y	z	x	y	z	x	y	z
t1	-4.15	14.84	4.30	-4.33	14.83	4.13	-4.41	14.81	4.14
t2	-6.22	14.36	3.32	-6.31	14.35	3.19	-6.37	14.35	3.10
t3	-7.27	14.04	2.43	-7.44	13.99	2.20	-7.58	13.94	2.02
t4	-10.29	12.25	-0.05	-10.21	12.32	-0.02	-10.13	12.39	0.02
t5	-10.59	11.09	-4.56	-10.36	11.36	-4.42	-10.07	11.73	-4.14
t6	-7.11	11.61	-8.34	-6.94	12.15	-7.68	-6.75	12.51	-7.29

Hip contact force (HCF) validation

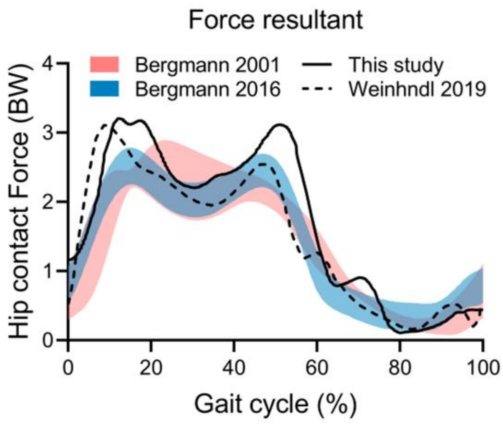

Figure S6. Comparison of the resultant HCF computed by means of the multibody model against *in vivo*[7,8] and *in silico*[9] findings reported in the literature.

References

1. Simtk-OpenSim. Gait 2392 and 2354 Models - OpenSim Documentation - Global Site. https://simtk-confluence.stanford.edu:8443/display/OpenSim/Gait+2392+and+2354+Models#Gait2392and2354Models-Musclegeometry (accessed on 30 November 2023).
2. Fessler, H. & Fricker, D. C. Friction in femoral prosthesis and photoelastic model cone taper joints. *Proc. Inst. Mech. Eng. Part H J. Eng. Med.* **203**, 1–14 (1989).
3. Dyrkacz, R. M. R. *et al.* Finite element analysis of the head–neck taper interface of modular hip prostheses. *Tribol. Int.* **91**, 206–213 (2015).
4. Falkenberg, A., Biller, S., Morlock, M. M. & Huber, G. Micromotion at the head-stem taper junction of total hip prostheses is influenced by prosthesis design-, patient- and surgeon-related factors. *J. Biomech.* **98**, 109424 (2020).
5. English, R., Ashkanfar, A. & Rothwell, G. A computational approach to fretting wear prediction at the head-stem taper junction of total hip replacements. *Wear* **338–339**, 210–220 (2015).
6. Elkins, J. M., Callaghan, J. J. & Brown, T. D. Stability and trunnion wear potential in large-diameter metal-on-metal total hips: A finite element analysis. *Clin. Orthop. Relat. Res.* **472**, 529–542 (2014).
7. Bergmann, G. *et al.* Hip forces and gait patterns from routine activities. *J. Biomech.* **34**, 859–871 (2001).
8. Bergmann, G., Bender, A., Dymke, J., Duda, G. & Damm, P. Standardized loads acting in hip implants. *PLoS One* **11**, 1–23 (2016).
9. Weinhandl, J. T. & Bennett, H. J. Musculoskeletal model choice influences hip joint load estimations during gait. *J. Biomech.* **91**, 124–132 (2019).
